# Diagnosis of Feline Food Sensitivity and Intolerance Using Saliva: 1000 Cases

**DOI:** 10.3390/ani9080534

**Published:** 2019-08-06

**Authors:** W. Jean Dodds

**Affiliations:** Hemopet, 11561 Salinaz Avenue, Garden Grove, CA 92843, USA; info@hemopet.org; Tel.: +1-714-891-2022 (ext. 115); Fax: +1-714-891-2123

**Keywords:** adverse food reactions, pet cats: saliva test

## Abstract

**Simple Summary:**

This study asked whether a novel saliva-based diagnostic test could predict food sensitivities and intolerances in cats. Clinical samples were obtained from 1000 cats proven or suspected to have adverse food reactions (AFR). Most were domestic shorthairs over 10 years old and weighed around 5 kg; they were equally distributed between spayed females and neutered males. Saliva was collected after at least an 8-h fast with a dental cotton rope, placed in a special saliva collection tube, and sent to the laboratory. Salivary reactions to 24 common foods were measured with immunological reagents. Low reacting foods were lamb, cow milk, pork, turkey, wheat (lowest) and white-colored fish, whereas high reacting foods were millet, white potato, rice (highest) and salmon. Thus, this novel salivary-based food sensitivity and intolerance test, described previously for dogs, also provided a reliable and clinically predictive alternative to other methods of measuring AFR in cats.

**Abstract:**

This prospective study assessed the efficacy of a novel saliva-based immunoassay of IgA- and IgM-antibodies in predicting feline food sensitivities and intolerances. Clinical samples were obtained from 1000 cats proven or suspected to have food intolerances. Most were of domestic shorthair breed type, over 10 years of age, and weighed around 5 kg; they were equally distributed between spayed females and neutered males. Saliva was collected after at least an 8-h fast with a dental cotton rope, placed in a double-sleeved saliva collection tube, and sent to the laboratory. Salivary antibodies elicited by 24 common foods were measured with goat anti-canine IgA and IgM. Low reacting foods were lamb, cow milk, pork, turkey, wheat (lowest) and white-colored fish, whereas high reacting foods were millet, white potato, rice (highest) and salmon. Thus, the novel salivary-based food sensitivity and intolerance test, described previously for canines, also provided a reliable and clinically predictive alternative to food elimination trials, serum-based food allergy testing, and skin patch testing in cats. Manufacturers of commercial cat foods and treats, as well as those making homemade diets and treats for cats, should consider avoiding the more highly reactive foods as determined by the present study.

## 1. Introduction

It is well-recognized that wholesome nutrition is the key to maintaining a healthy immune system and resistance to disease [1,2,3,4]. Commercial foods ingested by animals on a regular basis may not be balanced in terms of major nutrients, minerals and vitamins, and some continue to add chemicals to the final product to enhance its stability and shelf-life [1,4,5]. The prevalence of adverse food reactions (AFR) in clinical veterinary medicine is increasing at an alarming rate in pet animals, along with the plethora of available commercial foods and home-prepared diets [1,4,6,7,8]. Nutritional deficiencies, excesses, or imbalances as well as the presence of undeclared ingredients in commercial pet foods when combined with exposures to various chemicals, drugs and toxins present a continual immunological and cellular inflammatory challenge [4,5,6,7]. These challenges can suppress immune function and cause chronic diseases including obesity and cancers, especially in those animals genetically susceptible to immune dysfunction (immune deficiency, autoimmunity, allergies) [1,2,4,5]. Interactions between the gastrointestinal (GI) tract, microbiome and immune system have been an increasing focus of current research in people and animals [5,9,10,11].

Genetic differences between individuals lead to quantitative variations in dietary requirements for energy and nutrient needs, and to maintain health [1,3,4]. Also, genetic defects may result in inborn errors of metabolism that affect one or more pathways involving nutrients or their metabolites. Nutritional factors that play an important role in immune function include zinc, selenium and vitamin E, vitamin B_6_ (pyridoxine), and linoleic acid [1,4]. Deficiency of these compounds impairs both humoral as well as cell-mediated immunity, and requirements usually increase in geriatric individuals, because immune function and the bioavailability of nutrients generally wanes with aging. As with any nutrient, however, excessive supplementation can lead to significant clinical problems, many of which are similar to the respective deficiency states of these ingredients [1,4,12,13].

An important component of this need for nutritional balance and adequacy involves the increasing incidence of food sensitivity and intolerances not only in humans but also in the companion animals that share our lives [1,2,3,4,5,6].

## 2. Materials and Methods

### 2.1. Study Population

**Figure 1:**
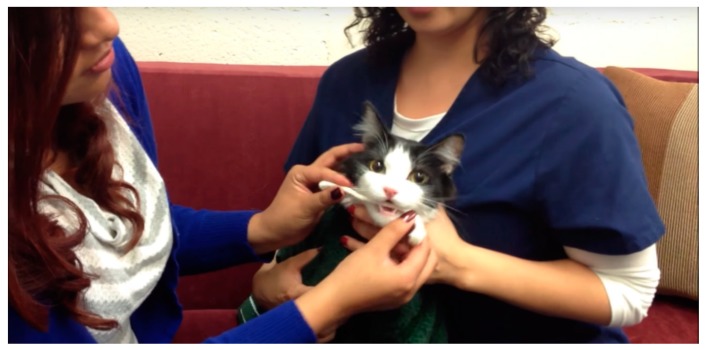
Collecting saliva from a cat for food intolerance testing.

Saliva from cats suspected of having food sensitivities or intolerances was submitted by veterinary clinics and their pet owner clients throughout North America; some samples came from overseas. The collection procedure used a thin cotton rope as described previously [2]. A total of 4214 feline samples have been received and analyzed by our laboratory since early 2013. For purposes of the present study analysis, 1000 cases were selected sequentially without preference covering the period of 21 July 2015 through 15 September 2018. The saliva collection method is shown in Figure 1.

### 2.2. Test Methodology

#### 2.2.1. Salivary Anti-IgA and IgM Diagnostics

The assays for salivary anti-IgA and anti-IgM, were performed using the specific ELISA Food Antigen–Coated Plates containing the 24 affinity-purified food antigens manufactured for this purpose (see Section 5). The detailed methodology has been previously described [1,2]. A result at or above 11.5 units/mL is considered to reflect food sensitivity and intolerance in both dogs and cats [2]. The obtained optical density (OD) readings were reported to 4 decimal places using an automated robotic analyzer. Those samples testing above the assay blank OD were then converted to the arbitrarily set units/mL standard for ease of reporting and comprehension [2]. The 24 foods tested in this assay included highly purified protein extracts of: beef, chicken, corn and cornstarch, duck, lamb and goat, cow milk, pork, soy, turkey, venison, wheat, white-colored fish and their oils, barley, hen eggs, lentils and peas, millet, oatmeal, peanuts and peanut oil, potatoes, quinoa, rabbit, rice, salmon and salmon oil, and sweet potatoes (see Section 5) [2]. This test methodology has been validated for sensitivity (93–99%), specificity (69–72%) and accuracy by likelihood ratios (3.08–5.30% for positive ratios; 0.63–0.65% for negative ratios) [2].

#### 2.2.2. Serum Anti-IgG Diagnostics

Serum samples were obtained at the time of saliva collection from 154 of the 1000 cases studied for the present analysis; reactivities were measured for the 24 foods tested using goat anti-canine IgG. None of these serum samples produced increased anti-IgG reactivities to any of the 24 foods tested.

## 3. Results

### 3.1. Population Demographics

Please see Table 1 and Table 2.

### 3.2. Food Reactivity Data

Table 3 shows the results in units/mL for the 24 foods tested, with either IgA- and/or IgM-antibodies, where the amounts exceeded the negative and weak reactive levels (i.e., greater than 11.5 units/mL) [1,2].

Table 4 shows the outcome test results of ten cases tested before and after removal of the identified reactive foods.

### 3.3. Case Study

**Figure 2:**
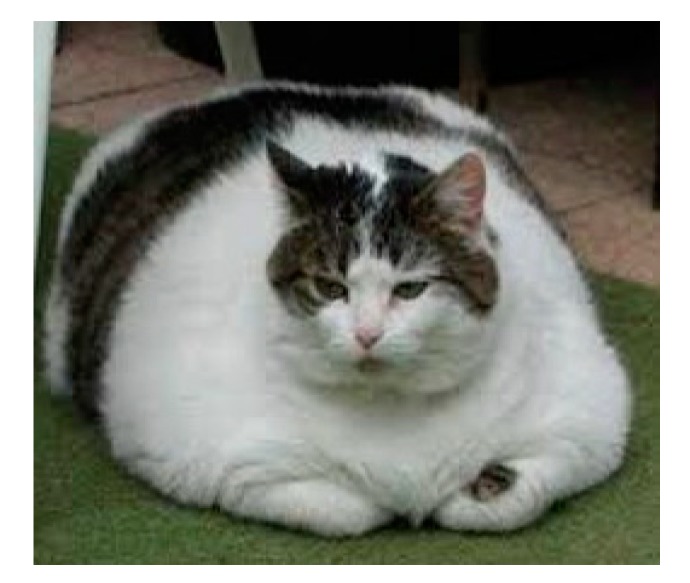
The obese cat.

“Bizz Bee”, 10-year-old Domestic Short Hair, neutered male cat weighing 9.5 kg (Figure 2). When initially tested for food intolerances, he was obese and ate primarily chicken and turkey, and had experienced reactions to fish. He had an itchy anal area and was scooting on carpets, soft stools and what was termed as “feline acne”. Food reactions were identified in his saliva to: Beef, chicken, corn and cornstarch, duck, white-colored fish and their oils, hen eggs, millet, peanuts and peanut oil, potatoes, quinoa, rabbit, rice, salmon and salmon oil, and sweet potatoes.

When these reactive foods were removed from his diet, except for chicken and turkey which the client elected to continue feeding, reactions were identified to: chicken, turkey, duck, lamb, soy, peanuts and peanut oil, rabbit, and sweet potatoes. He was still itchy and scratching and had ingested hair balls. Upon questioning the client, she was cooking with peanut oil, although sources of the other reactive foods were not apparent, except perhaps within the numerous supplements she fed him. Once the peanut oil was changed to safflower oil and supplements containing reactive ingredients and flavorings were removed, his itching and scratching ceased and his stools became firm.

## 4. Discussion

The published literature and clinical experience documents that cats, like dogs, can suffer from AFR, including chronic GI enteropathies and a variety of other clinical signs [4,8]. The difficulty, however, arises in attempting to accurately determine which food or foods are to blame [1,2,4,6,14]. While AFR have classically been diagnosed by skin patch or prick testing, food elimination trials, and measurement of serum IgD, IgE or IgG, published studies have shown that food elimination trials are fraught with discrepancies and failure of animal owners to comply, whereas skin patch or prick testing is unsightly [2,4]. Further, serum testing of IgG, IgA and IgM levels in companion animals to identify AFR is poorly predictive of manifested clinical issues [14,15,16]. These diagnostic dilemmas consequently raise the question of the accuracy and validity of the saliva-based test methodology used in the present paper. Regarding the use of goat anti-canine IgA and IgM reagents to measure the parallel antibody levels in cats, this was necessitated because the author’s prior testing of the available goat anti-feline reagents found them not sufficiently purified to avoid detection of contaminating proteins. Additionally, it is also well-recognized that the conserved epitopes of canine and feline antigens allow for tight cross-reactivity of antibody reagents with more than a 98%–99% identity [17]. There was no apparent loss of assay sensitivity using the anti-canine IgA and IgM antibodies in cats and thus the testing applies to both species with equivalent sensitivity. 

In defense of the present test method developed by this author for both dogs and cats, a previously published peer-reviewed study demonstrated the very high sensitivity, specificity, and positive likelihood ratios for the test [2]. Additionally, clinical outcome studies were completed for 50 canine cases and 15 cases received follow up retesting [2]. These results supported the clinical utility of the test. The present data for assessing feline AFR document parallel findings for anti-IgA and/or anti-IgM salivary testing in cats, including the results from 1000 cases (Table 1, Table 2 and Table 3) with outcome studies in ten cases (Table 4), and the above stated negative results for anti-IgG serum-based food reactivities of 154 of these cases. 

Anti-IgA testing in body secretions measures the level of secretory immunity released into body fluids such as saliva within the prior two years in dogs and cats [1,2,5,15,16]. Anti-IgM measurements reflect the body’s primary immune response mechanism to challenges of the prior 2–6 months [1,2,5,16]. While the results shown in Table 3 reflected reactivity levels using either IgA and/or IgM, the degree of food reactions for the 24 foods using both antibodies were similar. 

Other important factors impact upon the need for better diagnostics for AFR, especially in cats. Nutritional influences can have a profound effect on thyroid metabolism [1,2,12,13]. The classical example is the iodine deficiency that occurs in individuals eating cereal grain crops grown on iodine-deficient soil [1]. This will impair thyroid metabolism because iodine is essential for formation of thyroid hormones [1,12]. However, too much iodine, especially as found in fish-flavored cat diets, can be as harmful to thyroid function as too little, and has been previously determined to be a potential cause of clinical hyperthyroidism in older cats [12,13].

While AFR comprise about a third or more of the recognized GI diseases of both dogs and cats [1,2,3,4,5,6,7,17], unlike dogs, where gluten foods like wheat and barley are typically quite reactive on saliva-based and serum testing [4,5,6,8], wheat was the lowest tested food in the present cat studies. Similarly, white-colored fish tested very low (second lowest) whereas salmon tested much higher (Table 3). Also, in contrast to dogs [2], the highest stated food reactions in cats are to beef, fish, and chicken [7], although the present study found high results to rice (highest), millet (second highest), and white potatoes (Table 3). Rice, shown to be unsuitable here for cats, is commonly recommended as a hypoallergenic food for pets with food sensitivities or IBD [4,6,8]. As millet is a goitrogenic food, could older cats that have eaten and are eating millet be predisposed to hyperthyroidism like they are from iodine excess? [12,13]. Regardless, these findings indicate that manufacturers of commercial cat foods and treats, as well as those making homemade diets and treats for cats, should consider avoiding these more highly reactive foods as determined by the present study, and as described by others for feline canned diets and casein [18].

## 5. Patents

**Patents:** Issued US patents: 7,867,720; 7,892,763; 8,450,072; 8,450,074; Canadian patents 2,743,714; 2,771,948; and European patent 2,382,469.

**Endnotes:** NutriScan^®^, Hemopet, Garden Grove, CA 92843; www.nutriscan.org.

## Figures and Tables

**Table 1:** Summary of 1000 feline cases by breed type.

Parameter (%)	DSH *	DMH *	DLH *	Purebreds **
Average	26.5	2.3	3	See list below
Median	27	2	2	
Standard Deviation	2.8	1.7	2.3	
Range	24–31	0.5–4	0.7–4.5	

* Breed abbreviations: DSH, Domestic Short Hair; DMH, Domestic Medium Hair; DLH, Domestic Long Hair. ** Purebreds: Abyssinian, Persian, Maine Coon, Siamese, Ragdoll, British Shorthair, Russian Blue, Bengal, Bombay, Burmese, Sphynx, Manx, Tonkinese, Rex-Devon and Cornish, Himalayan.

**Table 2:** Summary of 1000 feline cases by age, sex and weight.

Parameter	Age (Yrs)	Sex SF (#)	Sex F (#)	Sex NM (#)	Sex M (#)	Weight (kgs)
Average	9.2	18.4	2.6	19.1	2.1	5.0
Median	9.3	18	2	19	2	4.9
Standard Deviation	0.86	3.87	1.81	4.06	1.71	0.31
Range	7.1–10.6	-	-	-	-	-

Sex abbreviations: SF, spayed female; F, female; NM, neutered male; M, male. # Number of cats by sex in the 24 microtiter assay plates.

**Table 3:** Summaries of food reactivities in 1000 feline cases of proven or suspected bowel disorders.

**(a) 12 Food Antigens**						
**Parameter (Units/mL)**	**Beef**	**Chicken**	**Corn**	**Duck**	**Lamb**	**Milk**
Average	12.43	19.24	12.67	9.1	4.33	5.1
Median	13	21	12	8	4	4
Std. Dev	6.4	7	6	4.5	2.9	2.7
**Parameter (Units/mL)**	**Pork**	**Soy**	**Turkey**	**Venison**	**Wheat**	White Fish *
Average	5.8	13.4	6.4	9.2	2.7	3.6
Median	5	14	7	10	2	3
Std. Dev	2.4	4.2	3.1	3.7	2.4	2.8
**(b) Another 12 Food Antigens**						
**Parameter (Units/mL)**	**Barley**	**Hen Egg**	**Lentil**	**Millet**	**Oatmeal**	**Peanut**
Average	9.9	7.4	9.1	24.3	15.5	12.2
Median	10	7	9	25	15	11
Std. Dev	4.3	3.5	3.8	5.9	4.7	5.1
**Parameter (Units/mL)**	**Potato**	**Quinoa**	**Rabbit**	**Rice**	**Salmon**	**Sweet Potato**
Average	23.2	19	14	25.9	20.9	17.1
Median	22	20	13	25	20	16
Std. Dev	6.5	6.7	4.5	6.9	6.1	6.1

* White Fish = any white-colored fish. Low reactors were lamb, cow milk, pork, turkey, wheat (lowest) and white-colored fish. High reactors were millet, white potato, rice (highest) and salmon.

**Table 4:** Outcome of 10 cases.

Case Number	Number Reactive Foods (Initial Test)	Number Reactive Foods (Follow Up Test) *
1–3	0	0
4	23	5
5	16	10
6	15	9
7	11	3
8	13	2
9	5	1
10	2	0
Total	85	30

* Reactive foods removed from diet; retested 2–6 months later.

## Abbreviations

AFR	Adverse food reactions
IBD	Inflammatory bowel disease
GI	Gastrointestinal
IgA	Immunoglobulin A
IgD	Immunoglobulin D
IgE	Immunoglobulin E
IgG	Immunoglobulin G
IgM	Immunoglobulin M
